# Treatment outcome of new smear positive pulmonary tuberculosis patients in Penang, Malaysia

**DOI:** 10.1186/1471-2334-14-399

**Published:** 2014-07-19

**Authors:** Muhammad Atif, Syed Azhar Syed Sulaiman, Asrul Akmal Shafie, Irfhan Ali, Muhammad Asif, Zaheer-Ud-Din Babar

**Affiliations:** 1 Discipline of Clinical Pharmacy, School of Pharmaceutical Sciences, Universiti Sains Malaysia, Penang, Malaysia; 2 Department of Pharmacy, The Islamia University of Bahawalpur, Punjab, Pakistan; 3 Discipline of Social and Administrative Pharmacy, School of Pharmaceutical Sciences, Universiti Sains Malaysia, Penang, Malaysia; 4 Respiratory Department, Penang General Hospital, Penang, Malaysia; 5 Department of Pharmacology, School of Pharmaceutical Sciences, Universiti Sains Malaysia, Penang, Malaysia; 6 Division of Pharmacy Practice, School of Pharmacy, University of Auckland, Auckland, New Zealand

**Keywords:** Smear positive pulmonary tuberculosis, Treatment outcomes, Unsuccessful treatment outcome, Tuberculosis treatment duration, Penang, Malaysia

## Abstract

**Background:**

According to the World Health Organization’s recent report, in Malaysia, tuberculosis (TB) treatment success rate for new smear positive pulmonary tuberculosis (PTB) patients is still below the global success target of 85%. In this study, we evaluated TB treatment outcome among new smear positive PTB patients, and identified the predictors of unsuccessful treatment outcome and longer duration of treatment (i.e., > 6 months).

**Methods:**

The population in this study consisted of all new smear positive PTB patients who were diagnosed at the chest clinic of Penang General Hospital between March 2010 and February 2011. During the study period, a standardized data collection form was used to obtain socio-demographic, clinical and treatment related data of the patients from their medical charts and TB notification forms (Tuberculosis Information System; TBIS). These data sources were reviewed at the time of the diagnosis of the patients and then at the subsequent follow-up visits until their final treatment outcomes were available. The treatment outcomes of the patients were reported in line with six outcome categories recommended by World Health Organization. Multiple logistic regression analysis was used to find the independent risk factors for unsuccessful treatment outcome and longer treatment duration. Data were analyzed using the PASW (Predictive Analysis SoftWare, version 19.0. Armonk, NY: IBM Corp).

**Results:**

Among the 336 PTB patients (236 male and 100 female) notified during the study period, the treatment success rate was 67.26% (n = 226). Out of 110 patients in unsuccessful outcome category, 30 defaulted from the treatment, 59 died and 21 were transferred to other health care facilities. The mean duration of TB treatment was 8.19 (SD 1.65) months. In multiple logistic regression analysis, risk factors for unsuccessful treatment outcome were foreign nationality, male gender and being illiterate. Similarly, risk factors for mortality due to TB included high-grade sputum and presence of lung cavities at the start of treatment, being alcoholic and elderly. Likewise, concurrent diabetes, presence of lung cavities at the start of the treatment and being a smoker were the significant predictors of longer treatment duration.

**Conclusion:**

Our findings indicated that the treatment success rate among the new smear positive PTB patients was less than the success target set by World Health Organization. The proportion of patients in the successful outcome category may be increased by closely monitoring the treatment progress of the patients with aforementioned high risk characteristics. Similarly, more aggressive follow-up of the treatment defaulters and transferred out patients could also improve the TB treatment success rate.

## Background

Tuberculosis (TB) is a public health problem with an annual incidence rate of about 8.6 million cases and an estimated 1.3 million deaths every year [1]. TB ranks as second major cause of adult mortality from an infectious disease worldwide, after human immunodeficiency virus (HIV) [1]. It has been estimated that between the years 2000 and 2020, approximately one billion people will be infected with Mycobacterium *tuberculosis*, 200 million infected people will develop active disease, and 35 million will die from TB globally if prevention and control plans are not further developed and executed [2]. Assessment of treatment outcome of newly diagnosed smear positive pulmonary tuberculosis (PTB) patients is used as a major pointer to gauge the effectiveness of national tuberculosis program (NTP). World Health Organization (WHO) has recommended that countrywide cohort analysis of TB treatment outcome should be performed every year. This strategy not only enables the NTP managers and staff to identify the problems but also allows them to take adequate and timely actions to improve the overall performance of the program [3-5].

According to WHO, TB treatment success rate among new smear positive PTB patients in Malaysia is still below the success target of 85% [1]. To date, a few studies from different states of Malaysia had reported the treatment outcome of TB patients with a very high heterogeneity in the results [6-8]. Neither of these studies had separately reported the treatment outcome of new smear positive TB patients, nor do any of these studies have established a link between patient characteristics and mortality during TB treatment. Furthermore, existing data are also lacking in terms of establishing a relationship between patient characteristics and duration of TB treatment.

Based on the WHO recommendations and the gaps in the existing literature, the aims of this study are to evaluate the treatment outcomes of new smear positive PTB patients as well as to determine risk factors for unsuccessful treatment outcome and longer treatment duration.

## Methods

### Study settings

The study was conducted at the chest clinic of Penang General Hospital (PGH). Penang General Hospital, with a capacity of 1,107 beds, is a point-of-reference tertiary health care facility in the Northern Region of Malaysia. The chest clinic of PGH is under the respiratory department with 70 beds [9]. In general, new PTB patients are not hospitalized.

At the chest clinic, a minimum of five to six medical officers manage routine patients with respiratory illnesses, whereby complex cases are managed by three full-time chest consultants. In addition to this, the chest clinic has dedicated paramedic staff to provide quality care to the patients. The TB diagnostic laboratory, which is situated adjacent to the chest clinic, has dedicated facility to investigate the specimens of suspected and existing TB patients using sputum smear examination, culture, nucleic acid amplification tests and drug sensitivity testing. The radiology and pathology departments of PGH also provide routine investigation services to TB patients.

Confirmed TB patients are advised to take their medication either at the chest clinic or at a primary health care unit. In general, patients are advised to take their daily medication under the direct observation of a staff nurse at the chest clinic or at a primary health care unit. However, some patients are allowed weekly packing of the daily dose. Weekly packing of the daily dose is only available at the chest clinic. Patients who continue their daily TB medication at a primary health care unit are advised to visit the chest clinic (every two weeks during the intensive phase and every month during the continuation phase) for routine investigation. Patients who default from their treatment for five consecutive days are traced by a team of staff comprising of a TB-coordinator, a staff nurse and an attendant [10].

### Study design and data collection

The population in this study consisted of all new smear positive PTB patients (N = 336) who were diagnosed at the study site between March 2010 and February 2011. A patient diagnosed with smear positive PTB as well as extrapulmonary tuberculosis (EPTB) was categorized as a case of PTB, and therefore was included in the study. The selection of this subgroup of patients was based on the recommendation of WHO to separately evaluate new smear positive PTB patients for the assessment of NTP’s performance [3].

During the study period, a standardized data collection form was used to obtain patient’s socio-demographic, clinical and treatment related data on age, sex, ethnicity, marital status, smoking habit, alcohol use, drug abuse, presence of any concurrent illness, site of disease, signs and symptoms of TB, chest X-ray findings, bacteriology results, duration of treatment and treatment outcome from his/her medical chart and TB notification form (Tuberculosis Information System, TBIS). Medical charts and notification forms of all the patients were reviewed at the time of their diagnosis and then at the subsequent follow-up visits until final treatment outcomes of all the patients were available (i.e., mid of December 2011). Some of the socio-demographic characteristics (i.e., employment status, monthly income and level of education) were obtained by interviewing the patients by a trained nurse [7,11].

During the treatment, the clinicians prescribed standard 6 months treatment regimen (2 months of isoniazid, rifampicin, pyrazinamide and ethambutol during intensive phase and 4 months of isoniazid and rifampicin during the continuation phase) to the patients [3]. However, for those patients who were still smear positive after 2 months of treatment and/or had cavitary disease at the start of the treatment received longer treatment with first-line anti-TB drugs [12-14]. As a result, 132 patients had 3 months of intensive phase, while continuation phase was prolonged (>4 months; maximum 7 months) in 141 patients (Note: a patient whose intensive phase was prolonged may or may not have prolonged continuation phase). Similarly, treatment duration also varied among those (n = 9) experiencing major side effects associated with anti-TB drugs [3].

The treatment outcomes of TB patients were reported according to six outcome categories as mentioned in the WHO guidelines [3]. These categories include; cured (a patient who has completed TB treatment with negative bacteriology result in the last month of the treatment and on at least one previous occasion), treatment completed (a patient who has completed TB treatment, but without the evidence of negative bacteriology result in the last month of the treatment and on at least one previous occasion), treatment failure (a patient in whom bacteriology result remains positive at five months or later), died (a patient who died due to TB or other cause during TB treatment), defaulter (a patient whose TB treatment was interrupted for two consecutive months or more after registration), transferred out (a patient who has been referred to another recording and reporting unit, and whose treatment outcome was not available).

Cured and treatment completed were further summarized as treatment success [3,4] whereas, treatment failure, died, defaulter and transferred out represented unsuccessful treatment outcome [11,15-18].

The study was approved by the Medical Research Ethics Committee (MERC), Ministry of Health, Malaysia (Registration ID: NMRR-10-77-5099; MERC reference: dim. KKM/NIHSEC/08/08/04P10-69).

### Definition of terms

#### Prolonged treatment duration

The total duration of TB treatment longer than 6 months represented prolonged treatment duration [3].

#### High-grade sputum

Sputum was graded according to the number of acid-fast bacilli (AFB) visible at the time of sputum smear microscopy. To simplify the grading categories, four grades were (i.e., scanty positive, 1+, 2+ and 3+) were combined to two categories. Scanty positive and 1+ represented low-grade sputum, whereby 2+ and 3+ denoted high-grade sputum [19].

### Statistical analysis

Data were analyzed using the PASW (Predictive Analysis SoftWare, version 19.0. Armonk, NY: IBM Corp). Simple logistic regression analysis was used to examine the possible association between the dependent variables (i.e., unsuccessful outcome, death and prolonged treatment duration) and selected socio-demographic and clinical variables. The variables which were statistically significant (i.e., p-value <0.05) in univariate analysis were entered into a multiple logistic regression analysis to predict the final independent factors. The adjusted odd ratios (AOR), 95% confidence interval (CI), beta, standard error and p-value were reported for each predictor. The model fit was assessed by chi-square, degrees of freedom and p-value. Pseudo R square values were reported to provide information about the percentage of variance explained by the model. A p-value of <0.05 was considered statistically significant [4,15,17].

## Results

During the study period, a total of 594 TB patients (all forms) were registered at the study site. However, 336 new smear positive PTB patients (56.57%) were included in the study. The mean age of the enrolled patients was 49.11 (SD 16.58) years. Table 1 provides the inclusive details about socio-demographic characteristics of the patients.

**Table 1:** Socio-demographic characteristics of the patients

**Characteristics**	**Patients n (%)**	**Characteristics**	**Patients n (%)**
**Sex**		**Drug abuser**	
Male	236 (70.24)	Yes	30 (8.93)
Female	100 (29.76)	No	306 (91.07)
**Age group (years)**		**Smoking status**	
≤24	30 (8.93)	Smoker	175 (52.08)
25–34	41 (12.20)	Non-smoker	161 (47.92)
35–44	56 (16.67)	**Alcoholism**	
45–54	77 (22.92)	Yes	115 (34.23)
55–64	71 (21.13)	No	221 (65.77)
65–74	37 (11.01)	**Employment status**	
≥75+	24 (7.14)	Employed	256 (76.19)
**Ethnicity**		Unemployed	80 (23.81)
Malay	104 (30.95)	Monthly income (MYR*)	
Chinese	179 (53.27)	≤1000	134 (39.88)
Tamil	33 (9.82)	1001–2000	136 (40.48)
Foreigners	20 (5.95)	2001–4000	48 (14.29)
**Marital status**		4001–6000	13 (3.87)
Single	90 (26.79)	>6000	5 (1.49)
Married	204 (60.71)		
Widow/divorced	42 (12.50)		
**Level of education**			
No education	28 (8.33)		
Primary	162 (48.21)		
Secondary	101 (30.06)		
University	45 (13.39)		

*Malaysian Ringgit.

Of the 336 study patients, approximately 97% patients had pulmonary disease, while only 2.98% were the confirmed cases of PTB and EPTB. The mean duration of TB treatment was 8.19 (SD 1.65) months. Table 2 provides a full description of the clinical characteristics of the patients.

**Table 2:** Clinical characteristics of the patients

**Characteristics**	**Patients n (%)**
**Form of TB**	
S^+^ PTB*	326 (97.02)
S^+^ PTB* and EPTB^†^	10 (2.98)
**Number of TB symptoms**	
≤2	95 (28.27)
3–4	169 (50.30)
≥5	72 (21.43)
**History of cough**	
<4 weeks	76 (22.62)
≥4 weeks	260 (77.38)
**Cough type**	
Productive	241 (71.73)
Non-productive	95 (28.27)
**X-ray lesions**	
Unilateral	143 (42.56)
Bilateral	193 (57.44)
**Lung cavities**	
No cavity	154 (45.83)
Single cavity	90 (26.79)
Two cavities	37 (11.01)
Multiple cavities	55 (16.37)
**Comorbidities**	
Diabetes	131 (38.99)
Hypertension	46 (13.69)
Acquired immunodeficiency syndrome	16 (4.76)
Other comorbidities	42 (12.50)

*Smear positive pulmonary tuberculosis; ^†^Extrapulmonary tuberculosis.

### Standardized treatment outcomes

Table 3 illustrates proportion of the patients in successful and unsuccessful treatment outcome categories. Out of 226 (67.26%) successfully treated patients, only 12.20% patients were classified as cured. In terms of unsuccessfully treated patients, 59 died, while 30 defaulted from the treatment. A relatively small proportion of the patients (n = 21) were transferred to other health care facilities.

**Table 3:** Treatment outcomes as per standard criteria

**Treatment outcomes**	**Patients n (%)**	**Total n (%)**
**Successful**		226 (67.26)
Cured	41 (12.20)	
Treatment completed	185 (55.06)	
**Unsuccessful**		110 (32.74)
Treatment failure	0 (0)	
Died	59 (17.56)	
Defaulter	30 (8.93)	
Transferred out	21 (6.25)	

### Predictors of unsuccessful treatment outcome

In multiple logistic regression analysis, the factors which remained significantly associated with unsuccessful treatment outcome were foreign nationality (AOR 21.24; 95% CI 4.61, 97.94), being illiterate (AOR 3.72; 95% CI 1.56, 8.87) and male gender (AOR 2.15; 95% CI 1.20, 3.87) (Table 4).

**Table 4:** Predictors of unsuccessful treatment outcome: multiple logistic regression analysis

**Independent variables**	***B***	***S.E***	***p-value***	**AOR (95% CI)**
Male	0.767	0.299	**.010**	2.15 (1.20, 3.87)
Chinese	-0.524	0.263	.057	0.59 (0.35, 1.07)
Foreigners	3.050	1.280	**<.0005**	21.24 (4.61, 97.94)
Illiterate	1.320	0.443	**.003**	3.72 (1.56, 8.87)

p-value of less than 0.05 in bold; Model summary (chi-square = 47.55, degrees of freedom = 4, p = <.0005, pseudo R^2^ = .132).

As described earlier, the proportion of patients died during TB treatment accounted for approximately 54% (i.e., 59 out of 110 patients) of the total patients in unsuccessful treatment outcome category. Therefore, further analysis was conducted to identify the factors which were significantly associated with the death of the patients.

In multiple logistic regression analysis, the factors which remained significantly associated with patients’ mortality were high-grade sputum at the start of treatment (AOR 11.19; 95% CI 2.50, 30.11), presence of lung cavities at the start of the treatment (AOR 7.25; 95% 2.76, 19.07), being alcoholic (AOR 2.54; 95% CI 1.17, 5.51) and being older (AOR 1.03; 95% CI 1.01, 1.06) (Table 5).

**Table 5:** Predictors of mortality: multiple logistic regression analysis

**Independent variables**	***B***	***S.E***	***p-value***	**AOR (95% CI)**
Male	0.580	0.523	.268	1.79 (0.64, 4.98)
Age*	0.027	0.013	**.036**	1.03 (1.01, 1.06)
History of ≥4 weeks cough	0.999	0.586	.088	2.72 (0.86, 8.57)
Lung cavities	1.980	0.493	**<.0005**	7.25 (2.76, 19.07)
High-grade sputum	2.420	0.765	**.002**	11.19 (2.50, 30.11)
Diabetes mellitus	0.701	0.372	.060	2.02 (0.97, 4.18)
Smoker	0.020	0.448	.965	1.07 (0.41, 2.36)
Alcoholism	0.934	0.394	**.018**	2.54 (1.17, 5.51)

p-value of less than 0.05 in bold; *Continuous variable; Model summary (chi-square = 71.71, degrees of freedom = 8, p = <.0005, pseudo R^2^ = .216).

### Predictors of prolonged treatment duration

In multiple logistic regression analysis, determinants of prolonged treatment duration (i.e., more than 6 months) were concurrent diabetes (AOR 6.80; 95% CI 2.98, 15.51), presence of lung cavities at the start of the treatment (AOR 2.67; 95% 1.29, 5.77) and being a smoker (AOR 2.43; 95% 1.18, 5.03) (Table 6).

**Table 6:** Predictors of prolonged treatment duration: multiple logistic regression analysis

**Independent variables**	***B***	***S.E***	***p-value***	**AOR (95% CI)**
Unemployment	-0.951	0.398	**.017**	0.39 (0.18, 0.84)
Lung cavities	0.981	0.375	**.009**	2.67 (1.29, 5.57)
High-grade sputum	0.662	0.371	.074	1.94 (0.94, 4.0)
Diabetes mellitus	1.920	0.421	**<.0005**	6.80 (2.98, 15.51)
Smoker	0.890	0.371	**.016**	2.43 (1.18, 5.03)

p-value of less than 0.05 in bold; Model summary (chi-square = 49.38, degrees of freedom = 5, p = <.0005, pseudo R^2^ = .212).

## Discussion

In our study, TB treatment success rate among new smear positive PTB patients was 67.26%. This was less than the target success rate for new smear positive PTB patients. According to a recent report, the TB treatment success rate in Malaysia is declining [20]. One of the possible reasons for this decline over the past few years could be the influx of increasing number of people from neighboring countries seeking employment in Malaysia. Studies showed that foreigners either prefer to be transferred to their countries or defaulted from the treatment due to the fear of compulsory expulsion by the immigration authorities [15,21]. Similar to the findings of our study, two African studies also reported low treatment success rate in TB patients [17,22]. Likewise, in recent years, TB treatment success rate (new smear positive PTB cases) in Japan, Hong Kong and Fiji was less than 70%. Similarly, other developed countries from Western Pacific Region like Australia, New Zealand and Singapore showed a treatment success rate of less than 80% in new smear positive PTB patients [1].

As described in the Results section, one of the major reasons for non-attainment of success target was the higher death rate (17.56%) among the patients. Published data from the United States and Western Pacific Region showed that the death rates among TB patients ranged from 14–21% [1,23,24]. Ditah et al stated that in some of the deaths, TB is only incidental and not causal. Furthermore, they emphasized that it is unreasonable to consider such deaths as unsuccessful treatment outcome of TB [11]. Likewise, a study from Russia further highlighted that using such deaths during TB treatment as an indicator of NTP’s performance might be misleading, and that these deaths might not have been preventable even with improvements in the TB services [25]. Studies from the United Kingdom and Switzerland reported that WHO’s target of 85% treatment success rate was missed mainly due to a higher proportion of deaths, mostly due to causes other than TB [5,26]. Consequently, based on the reasoning and recommendations given in the above cited studies, the treatment success rate in the present cohort analysis could be raised to 73.85% (by excluding 30 patients from the final analysis in whom TB was incidental to death, as mentioned in the mortality review form which was completed by the mortality review panel). Indeed, the application of modified criteria appears to be logical to permit a more objective evaluation of Malaysian NTP. However, because the WHO outcome criteria recommend reporting the outcome for a complete cohort, the figure 67.26% is appropriate for international comparison purposes.

In this study, defaulters were the second most common group of patients in the unsuccessful treatment outcome category. Our finding (8.93%) is somewhat comparable to the results of studies from Singapore (9%), Germany (10%), Cameron (20%) and Libya (28%) [4,17,22,27]. In contrast, American and Norwegian studies showed relatively lower defaulter rates among TB patients [15,28]. This inconsistency in the findings might be the consequence of differences in patient characteristics or as a result of varying criteria of NTP to trace and follow-up the treatment defaulters.

In the transferred out group, most of the patients were foreigners and the ones who left the country. It is not compulsory for the Malaysian immigration authorities to expel the foreigners who are diagnosed with active TB. However, usually the clinicians encourage sending such patients to their home countries for further treatment. In the present study, 12 out of 20 foreigners were transferred to their countries. However, the information about their treatment outcome was not available. Similarly, two foreigners defaulted from the treatment, possibly due to the fear of compulsory expulsion from the country. Lack of follow-up of the transferred out patients was unfortunate, since these patients might conceal their illness for the sake of living and securing their jobs in Malaysia.

One of our study’s most important finding was the attainment of low cure rate (12.20%) which was attributable to the unavailability of sputum specimen for microscopy during the last month of treatment. According to WHO, a PTB patient can be declared as cured if he/she has documented evidence of treatment completion with negative bacteriology result in the last month of treatment and on at least one previous occasion [3]. In actual practice, meeting this criterion is quite difficult. Usually after regular intake of anti-TB drugs for two to three months, patients are unable to produce sputum throughout the remaining period of their treatment. As a result, sputum specimen is not available for bacteriology. Possible solutions to cope with this challenge are sputum induction, gastric aspirates or bronchoalveolar lavage [18] at set time points suggested by WHO [3].

Studies from the United Kingdom also reported a low cure rate owing to difficulties in obtaining sputum specimens during the last month of the treatment [11,28]. Along the same lines, a recent global TB report showed low cure rates among PTB patients in Australia and New Zealand [1]. On the other hand, studies from Singapore, Germany and Norway showed higher cure rates among new smear positive PTB patients [4,15,27]. None of these studies provided information about the sputum induction during the last month of treatment. However, a Swiss study reported when spontaneous sputum was not available from the patients, a chest therapist induced sputum during the last month of TB treatment [16].

In multiple logistic regression analysis, male gender, illiteracy and foreign nationality were the independent predictors of unsuccessful treatment outcome. In comparison to Malaysians, foreigners had about 21 times higher chance of unsuccessful treatment outcome. One of the major reasons for this finding was the preferred expulsion of infected foreigners to their home countries. Likewise, illiteracy among the study patients was the second highest predictor (OR 3.72) of unsuccessful treatment outcome. A Malaysian study found a positive relationship between TB-related knowledge and education level of the patients [29]. Low level of TB-related knowledge might be a stronger barrier in completion of TB treatment. It could be assumed that this subgroup of people might stop taking anti-TB drugs upon resolving the TB-related signs and symptoms. With regard to gender, males had 2.18 times higher probability of unsuccessful treatment outcome when compared with females. Earlier studies also reported a direct association between male gender and the risk of unsuccessful TB treatment outcome [4,28]. Higher probability of unsuccessful outcome in men might be attributed to their high risk behaviors such as alcohol, substance and tobacco abuse [7].

We found that the risk factors for the patients’ mortality due to TB were high-grade sputum and presence of lung cavities at the start of treatment, alcoholism and older age. Consistent with our findings, studies from Turkey and China showed that extensive lung involvement was associated with higher mortality rate among PTB patients [30,31]. In contrast, two American studies demonstrated that the extent of lung involvement was negatively associated with patient death possibly due to the fact that the presence of cavities might raise the probability of an early diagnosis of TB in a patient [23,24]. When compared with non-alcoholics, alcoholics had 2.54 times higher probability of dying from TB. Our finding is similar to the findings of a study which showed a positive association between alcohol use and mortality in TB patients [4]. Studies showed that older patients are less likely to complete their treatment due to death [25,28]. Common of these studies, this study also showed that older patients had a greater probability of dying during their TB treatment.

With regard to the treatment duration, concurrent diabetes, presence of lung cavities at the start of TB treatment and smoking were independent determinants of longer treatment duration. Diabetes and smoking can weaken the body’s immune system by affecting the cell-mediated immunity [10,32,33]. Weakening of immune system could delay the sputum conversion time and thus the treatment duration may be prolonged. With regard to the cavitary disease, available literature confirmed that patients exhibiting lung cavities at the time of their TB diagnosis had longer treatment duration owing to extended sputum conversion time [16]. Likewise, this study also showed that the probability of longer treatment duration was 2.67 times higher in patients with cavitary disease compared with those who had no evidence of lung cavities at the time of TB diagnosis.

Interestingly, in our study, unemployed patients had 2.56 times higher chances of completing their TB treatment in 6 months. One possible justification for this finding might be that because of depression over being jobless, unemployed patients visited the clinic more frequently to seek health care, and thus enabled the clinicians to closely monitor the progress of treatment.

## Conclusion

In this study, the TB treatment success rate was less than the success target set by WHO. The cure rate was only 12.20% because most of the patients did not report sputum production during the last month of their TB treatment. The lower treatment success rate was attributed to a higher proportion of the patients in died and defaulter treatment outcome categories. In addition, 6.25% of patients in the transferred out category also led to a lower treatment success rate.

The proportion of patients in successful outcome category could be improved by closely monitoring the clinical improvement in the patients with identified risk factors. Similarly, more aggressive follow-up of treatment defaulters and transferred out patients could also improve the TB treatment success rate. The clinicians may encourage the foreigners to complete their treatment in Malaysia. Perhaps, this measure could improve the treatment success rate.

To improve the cure rate, during the last month of TB treatment, the clinicians should consider inducing sputum in non-sputum producing patients. Finally, though TB treatment outcome criteria set by WHO appears to be clear and inclusive, there are limitations, and require further improvements to permit an objective evaluation of NTP.

### Study limitations

The results of our study should be applied with caution to evaluate the overall TB treatment success rate in Malaysia. Because of industrial setup and tourism industry, Penang has become multicultural state. As a result, large number of foreigners and Malaysian citizens from other parts of country are settled in Penang. Doubtless, if diagnosed with active TB, these subsets of people are more likely to have unsuccessful treatment outcome (i.e., defaulter and/or transferred out). Secondly, PGH is a referral hospital for the Northern region of Malaysia. Therefore, it is assumed that relatively complex TB patients consulted or were referred to PGH. Indeed, this fact is further supported by a higher proportion of the patients in the death category of treatment outcomes.

We conducted our study in Penang based on the recommendations of WHO to separately evaluate TB treatment outcome at peripheral, district, and state levels [3]. Indeed, this strategy identifies the states or units which are performing well and thus enables the NTP staff to replicate successful practices elsewhere.

## Competing interests

The authors declare that they have no competing interests.

## Authors’ contributions

MAT has significant contribution in data acquisition, data analysis & interpretation and writing of the manuscript. MAT, SASS, AAS, MAS and ZB have substantial contribution in conception and design of study. MAS, AAS, SASS, IA and ZB have revised intellectual content of the manuscript. AAS, MAS and IA have contributed in data acquisition and manuscript drafting. Final version of manuscript is approved by all authors. All authors act as the guarantor for the overall content.

## Pre-publication history

The pre-publication history for this paper can be accessed here:

http://www.biomedcentral.com/1471-2334/14/399/prepub
